# Low-Temperature Dried Alginate/Silica Hybrid Aerogel Beads with Tunable Surface Functionalities for Removal of Lead Ions from Water

**DOI:** 10.3390/gels11060397

**Published:** 2025-05-27

**Authors:** Jiuqi Wei, Shilong Yang, Zhicheng Zhu, Jialu Lu, Bencong Zhang, Mingmei Zhang, Wei Wei

**Affiliations:** 1 School of Chemistry and Chemical Engineering, College of Energy and Power Engineering, Public Experiment and Service Center, Jiangsu University, Xuefu Road 301, Zhenjiang 212013, China; 2 Advanced Analysis and Testing Center, Nanjing Forestry University, Longpan Road 159, Nanjing 210037, China; yshl6072@163.com; 3 Shanghai Key Laboratory of Special Artificial Microstructure and Technology, School of Physics Science and Engineering, Tongji University, Siping Road 1239, Shanghai 200092, China

**Keywords:** aerogel beads, sol–gel, hybrid materials, adsorption, lead ions

## Abstract

Low-temperature dried alginate/silica hybrid aerogel beads with a large specific surface area (160.8 m^2^/g), low density (0.160 m^2^/g), and high degree of sphericity were successfully fabricated. Single networks of silica aerogels beads were synthesized via by calcining hybrid aerogel beads in air. Moreover, alginate-derived carbon/silica aerogel beads were also obtained by the thermal treatment of the hybrid aerogel beads in nitrogen, which were indicative of the double networks of the as-synthesized crack-free hybrid aerogel beads for the first time. The adsorption performances of above aerogel beads were also investigated. Meanwhile, using a common silane coupling agent as a modifying agent, a series of hybrid aerogel beads with tunable functional surfaces were obtained. The results showed that the obtained samples adsorbed Pb^2+^ well, and the hybrid aerogel beads modified with KH-590 exhibited an experimental maximum adsorption capacity of Pb^2+^ of 193.73 mg·g^−1^.

## 1. Introduction

Aerogel materials have gained considerable interest in recent years as a new state of matter, owing to their dual structural nature comprising nanoscale skeletons and macroscopic features [1]. However, the poor mechanical properties of inorganic aerogels (such as silica aerogels) hampered the development of inorganic aerogels. Recently, many studies have addressed the potential use of insoluble polysaccharides as building blocks for the fabrication of enhanced silica aerogel materials, such as flexible and translucent cellulose–silica aerogels [2], freeze-dried nanofibrillated cellulose aerogels [3], and polysaccharide-based aerogel microspheres [4]. Previous studies have shown that a wide range of polysaccharides are available for aerogel fabrication (e.g., chitosan, cellulose, and pectin) [2,3,4]. Among polysaccharides, alginate stands out as a versatile and eco-friendly candidate due to its ionic crosslinking ability, biocompatibility, biodegradability, and abundant carboxylate groups that can interact with cations [5,6,7]. With the development of aerogel preparation technology, synthesizing silica hybrid aerogels with special and improved properties have been considered as a rational process to expand the broad range of applications of aerogels.

In particular, hybrid aerogels with a spherical shape as the main morphology are porous materials that can be exploited in drug delivery and environmental remediation. Yu and co-workers reported the formation mechanism of graphene oxide/chitosan aerogel microspheres by combining electrospraying with the freeze casting method [8]. Zhang et al. prepared silica aerogel microspheres via ambient pressure drying [9]. The novel macroscopic structure and low-cost preparation of hybrid aerogels are issues in aerogel research requiring urgent addressal. Our research group has reported aerogel bead adsorbents exhibiting free separation, and these adsorbents exhibit macroporous network structure, resulting in superior and excellent adsorption performance [10,11]. In the field of adsorbent research, hybrid aerogels were also developed for the removal of organic dyes and heavy metal ions [12,13,14,15,16]. The results show that their multilayer morphology and easily regulated pore structure creates more opportunities for them in the field of adsorption. In particular, surface modification technology plays a key role in influencing the adsorption properties of aerogel materials. Meanwhile, great efforts have been taken in the modification of hybrid aerogels to adapt to water treatment applications and to even enhance their selective absorption and separation performance [17]. Ashori et al. developed a method for enhancing the oil adsorption capacity of cellulose nanofibers by modifying their surface using hexadecyltrimethoxysilane [18]. Qin et al. prepared functional aerogels crosslinked with hydrogen bonds and reported their hydrophobic and lipophilic properties [19]. The modification technology can produce a material with different surface properties that can overcome the performance limitations of a single material. However, to the best of our knowledge, there are no reports on the synthesis of low-temperature dried alginate/silica hybrid aerogel beads and their performance in adsorption application.

In this study, hybrid aerogel beads were prepared using the orifice-coagulation bath (OCB) method under ambient pressure drying (APD) using sodium alginate as a raw material. The selection of cationic species and crosslinking methodology exerts significant influence on the structural and functional characteristics of alginate-based materials. Among various approaches, ionic crosslinking with divalent cations, particularly calcium ions (Ca^2^⁺), has emerged as the predominant technique due to its operational simplicity and biocompatibility [6]. The hybrid aerogel beads were obtained by structurally reinforcing the easily moldable polymer with SiO_2_. This modification solves the problem of insufficient strength of pure organic polymers. On the other hand, it improves the shape and toughness of amorphous silica. Moreover, due to the surface modification by common silane coupling agents (3-aminopropyl triethoxysilane, KH550; γ-methacryloxypropyl trimethoxy silane, KH580; 3-mercaptopropyl trimethoxysilane, KH590), a series of functionalized hybrid aerogel beads were prepared. The physicochemical properties and adsorption behavior of the functionalized hybrid aerogel beads were studied to provide experimental and theoretical basis for the application of hybrid aerogel beads in the rapid treatment of heavy metals.

## 2. Results and Discussion

### 2.1. Synthesis of Hybrid Aerogel Beads

**Figure 1:**
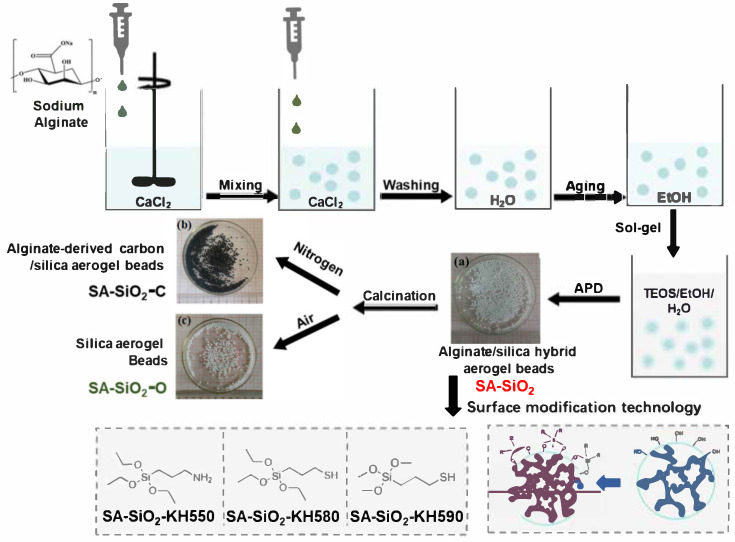
Schematic diagram of low-temperature dried alginate/silica hybrid aerogel beads with tunable functional surfaces.

The alginate/silica hybrid aerogel bead adsorbents were synthesized using a calcium ion retardation method in the orifice-coagulation bath, as shown in Figure 1 [11]. Subsequently, different silane coupling agents were used to modify the surface of the hybrid aerogel beads to obtain hybrid aerogel adsorbents with different surface groups. Meanwhile, the calcination strategy was adopted to obtain aerogel adsorbents with different skeletons (alginate-derived carbon/silica aerogel beads, SA-SiO_2_-C, and silica aerogel beads, SA-SiO_2_-O). The brittle SA-SiO_2_ network interwoven with the tough SA-Ca^2+^ network promotes their uniform mutual penetration [14]. This synergistic effect has a significant impact on improving the viscoelasticity and mechanical strength of hydrogel beads. Of note is that the hydrogel beads containing pure physical cross links are constructed without the use of reagents such as catalysts and initiators [15]. Therefore, the low-temperature dried gel prepared with this method can be regarded as “green” and completely biodegradable.

### 2.2. Physical and Chemical Properties of Hybrid Aerogel Beads

**Figure 2:**
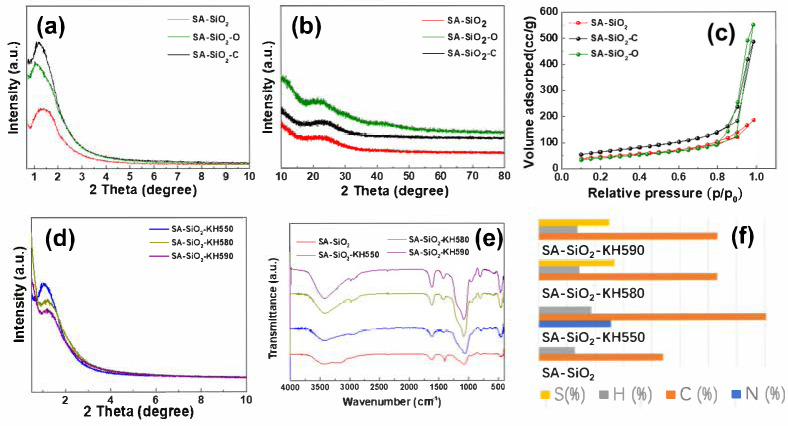
Small-angle X-ray diffraction (SXRD, (**a**,**d**)), X-ray powder diffraction (XRD, (**b**)), N_2_ adsorption–desorption isotherms (**c**), Fourier transform infrared spectroscopy (FTIR, (**e**)) spectrum, and element content chart patterns of low-temperature dried alginate/silica hybrid aerogel beads, the mass percentages of the elements S, H, C, and N in the samples (**f**).

Figure 2a shows the small-angle X-ray diffraction (SXRD) patterns of three aerogel beads (SA-SiO_2_, SA-SiO_2_-O, and SA-SiO_2_-C). It shows that a single peak appeared in the low diffraction angle region of 1–2° of SA-SiO_2_, and with the increase in the concentration of SiO_2_, the single peak became more obvious, indicating that, with the calcination process, the mesoporous structure of the aerogel became more obvious. This is because more monomers were used to construct three-dimensional (3D) networks after the calcination process, and the decomposition of organic substances in high-temperature calcination formed more mesopores in the aerogel formation process [20]. Meanwhile, the scattering intensity and position of the SXRD patterns of SA-SiO_2_-KH550 and SA-SiO_2_-KH580 remained almost unchanged, as shown in Figure 2d. This indicates that the thiol modification process did not affect the microstructure of the aerogel beads. However, peaks of SA-SiO_2_-KH550 shifted, while their intensities were different. A slight but measurable shift suggests local lattice distortions caused by KH550’s aminopropyl groups (–NH_2_) [21,22].

The XRD patterns (Figure 2b) show that different calcination environments (nitrogen/SA-SiO_2_-O and air/SA-SiO_2_-C) had little influence on aerogel beads. The pronounced peak broadening observed in XRD profiles originates predominantly from topological disorder inherent to amorphous architectures, as opposed to contributions from finite crystallite dimensions. Figure 2c shows the N_2_ adsorption–desorption isotherms of SA-SiO_2_, SA-SiO_2_-O, and SA-SiO_2_-C. It can be seen that the adsorption–desorption isotherms of the hybrid spherical aerogel were type IV isotherms, which are super-large-pore mesoporous solids with a wide pore size distribution. The nitrogen adsorption isotherms of SA-SiO_2_, SA-SiO_2_-O, and SA-SiO_2_-C aerogels indicate that their specific surface areas were 160.8 m^2^·g^−1^, 154.4 m^2^·g^−1^, and 210.8 m^2^·g^−1^, respectively. The experimental results show that a hierarchical aerogel can be prepared successfully through the simple carbonization of alginate aerogel at high temperatures [23].

SA-SiO_2_ showed a small shrinkage after the heat treatment process (Figure 1), due to the carbonization or the complete removal of the organic framework in SA. This is because the removal of part of the framework leads to a decrease in the overall strength of the framework [24]. SA-SiO_2_-C continued to exhibit a spherical structure after calcination in air. It indicates that the original framework structure of SA-SiO_2_ did not collapse after the carbonization process.

The functional groups of aerogel beads were determined using Fourier transform infrared spectroscopy (FTIR) [11]. Figure 2e displays the FTIR spectra of low-temperature dried alginate/silica hybrid aerogel beads. For all aerogel beads, peaks at 800 cm^−1^ are attributed to the bending vibration of Si–O, and peaks near 1073 cm^−1^ correspond to the stretching vibration of Si–O–Si [11]. The typical characteristic absorptions at 1618 cm^−1^ and 1425 cm^−1^ are attributed to the asymmetric and symmetric stretching of the C=O conjugated system of the alginate. Moreover, the stretching vibration of N-H overlapped the O-H stretching at around 3456 cm^−1^, indicating the alginate base amine functionality [11,25]. However, for SA-SiO_2_-KH580 and SA-SiO_2_-KH590, a weak absorption peak was observed around 2550 cm^−1^ due to the weak S-H stretching, which confirmed the presence of thiol groups in SA-SiO_2_-KH580 and SA-SiO_2_-KH590 [26]. Thus, surface modified aerogel beads can be successfully prepared by cross-linking the coupling agent with alginate. Figure 2f shows the mass ratio of elements contained in different functionalized low-temperature dried alginate/silica hybrid aerogel beads (Si is the calculated value). The nitrogen and sulfur contents in the low-temperature dried alginate/silica hybrid aerogel beads were significantly changed, indicating that the amino/thiol groups were well grafted onto the surface of the spherical aerogels.

**Figure 3:**
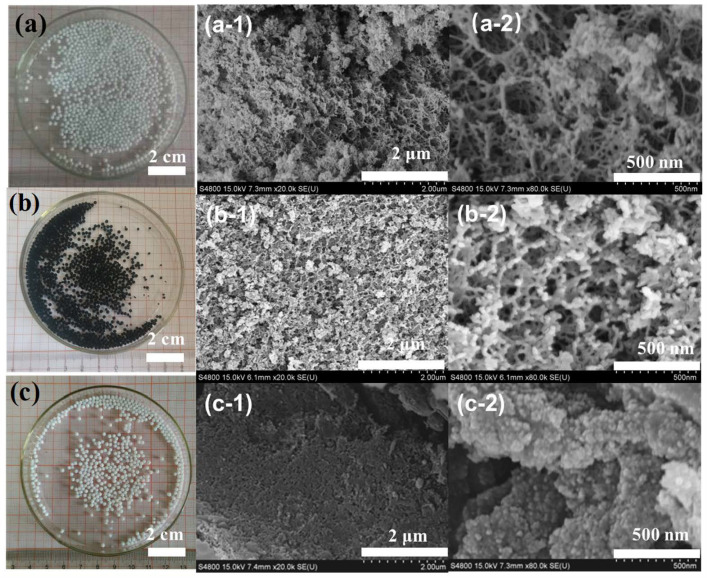
Photo and SEM images of SA-SiO_2_ (**a**,**a-1**,**a-2**), SA-SiO_2_-C (**b**,**b-1**,**b-2**), and SA-SiO_2_-O (**c**,**c-1**,**c-2**).

Figure 3a,b,c show the optical and SEM images of the low-temperature dried alginate/silica hybrid aerogel beads before and after heat treatment. In terms of appearance and form, the small beads calcined in a nitrogen atmosphere were black with no obvious cracks, had a shrinkage rate of ~18%, and had a relatively high sphericity. This means that the polysaccharides in the hybrid SA-SiO_2_ underwent carbonization and transformed into black carbon, and the small beads appeared black [23,24]. The aerogel calcined in an air atmosphere was milky white, without obvious cracks, and with a shrinkage rate of ~25% and a relatively high sphericity. This is because, in an air atmosphere, the polysaccharides in the hybrid SA-SiO_2_ are oxidized and removed; thus, the small beads appear milky white.

Figure 3 shows the scanning electron microscopy images (a1-2, b1-2, c1-2) of the samples SA-SiO_2_, SA-SiO_2_-C, and SA-SiO_2_-O. SiO_2_ presented a nanofilm-like porous structure both before and after heat treatment, and it was a typical porous material. Among them, SA-SiO_2_-C formed a smaller network structure than SA-SiO_2_. This was because the polysaccharide network structure of SA-SiO_2_ changed during the calcination process, and the network skeleton became slenderer. The SA-SiO_2_-O particles had a higher degree of dispersion and were more uniform. Their structure was quite different from the structure of conventional silica aerogel and was similar to that of porous materials prepared using the template method. The difference was that the silicon oxide material constructed using this method was in the form of nano-filaments, which is different from the form of conventional small silicon oxide particles.

**Figure 4:**
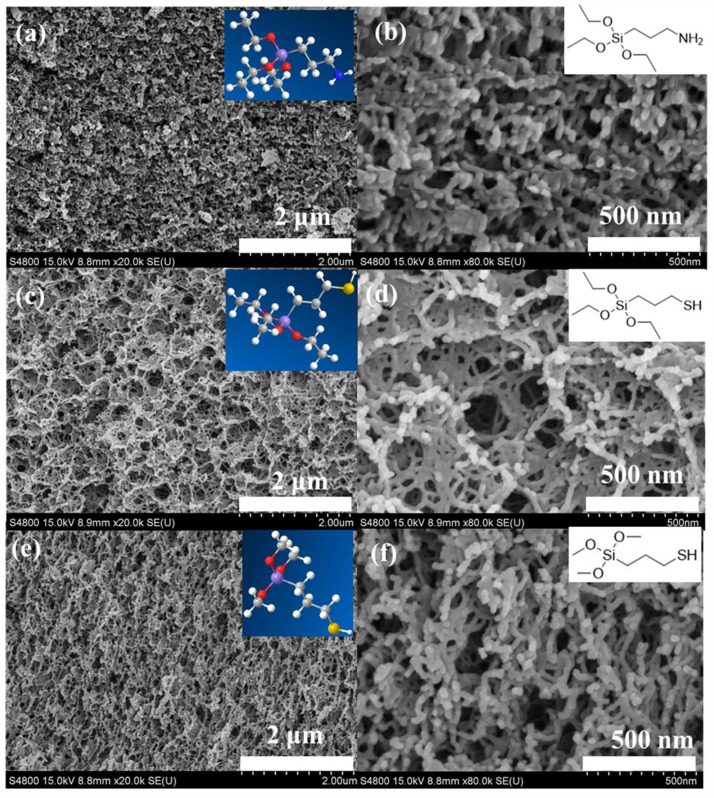
SEM images of SA-SiO_2_-KH550 (**a**,**b**), SA-SiO_2_-KH580 (**c**,**d**), and SA-SiO_2_-KH590 (**e**,**f**).

Figure 4 shows the scanning electron microscopy images of the samples SA-SiO_2_-KH550, SA-SiO_2_-KH580, and SA-SiO_2_-KH590. The microstructure morphology of the prepared samples was similar, all presenting nano-filamentous porous structures. Compared with the unfunctionalized sample SA-SiO_2_ (Figure 3(a-1)), the functionalized hybrid spheres had a denser network structure [25]. This is because, during surface modification, the silane coupling agent participated in the reaction, forming more network structures; thus, their network was denser. The experimental results of the packing bed density, shape, and appearance of the all-aerogel beads are given in Table 1.

The fabrication of hybrid aerogels beads involves three critical steps: (1) hydrogel preparation, (2) silicon-based structural skeletons incorporation [27], and (3) post-drying treatments. Among these processes, the introduction of silicon-based skeletons represents the most crucial step for obtaining intact hybrid spherical aerogel structures.

The incorporation of rigid silicon-based skeletons serves two essential functions: First, it effectively mitigates organic matrix shrinkage during ambient pressure drying. Second, it significantly enhances the mechanical strength of the resultant aerogel. This structural reinforcement is primarily achieved through the controlled hydrolysis and polycondensation of TEOS. The resulting hybrid structure consists of an interconnected organic silica network that demonstrates remarkable resistance to structural collapse during the drying process.

### 2.3. Adsorption Properties of Hybrid Aerogel Beads

Simulated wastewater was prepared with lead ion contents of 2 mg/L, 4 mg/L, 6 mg/L, 8 mg/L, and 10 mg/L. The standard curves were mapped using atomic absorption spectrometry, and the content of Pb^2+^ in the simulated wastewater after adsorption was calculated using this standard curve. The standard curve of Pb^2+^ was obtained based on the measurement results of atomic absorption spectroscopy [28]. The working curve equation is A = 0.00815c + 0.00033, R^2^ = 0.9998.

**Figure 5:**
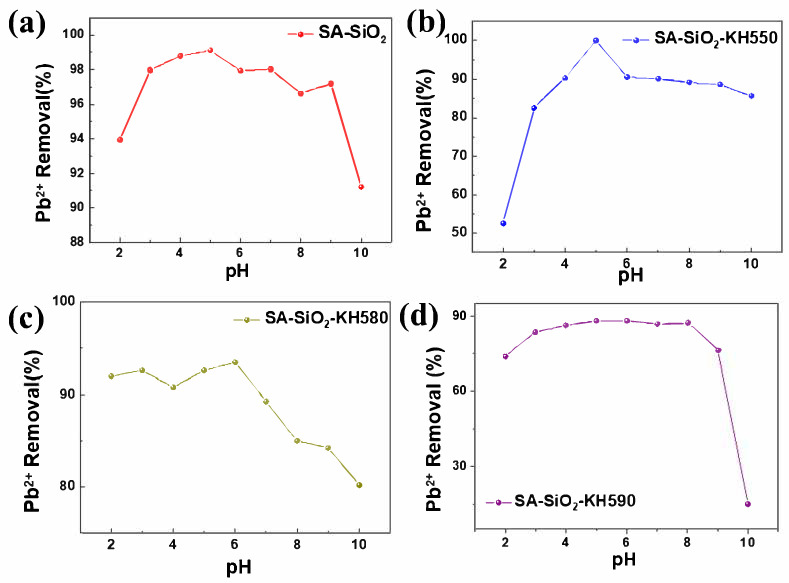
Effect of pH on adsorption of Pb(II) ion by alginate/silica hybrid aerogels: SA-SiO_2_ (**a**), SA-SiO_2_-KH550 (**b**), SA-SiO_2_-KH580 (**c**), and SA-SiO_2_-KH590 (**d**).

The pH of the solution is an important factor affecting adsorption [29,30], as it can not only change the state of charge on the surface of the adsorbent and influence the binding sites between metal ions and the adsorbent but also affect the existence state of metal ions [30]. The experiment investigated the adsorption of lead ions by four kinds of hybrid aerogels (SA-SiO_2_, SA-SiO_2_-KH550, SA-SiO_2_-KH580, and SA-SiO_2_-KH590, Figure 5) under different pH (2–10) conditions of the initial solution. The dosage of the fixed adsorbent was 20 mg, 20 mL of lead-containing simulated wastewater solution (10 mg/L) was used, and oscillation was carried out for 12 h. A pH at which the maximum percentage removal of Pb is observed is known as the optimal adsorption pH [29,30], as seen in Figure 5. Ultimately, in this experiment, pH = 5.0 was selected as the optimal adsorption pH for the adsorbents SA-SiO_2_ and SA-SiO_2_-KH550, and pH = 6.0 was selected as the optimal adsorption pH for the adsorbents SA-SiO_2_-KH580 and SA-SiO_2_-KH590.

**Figure 6:**
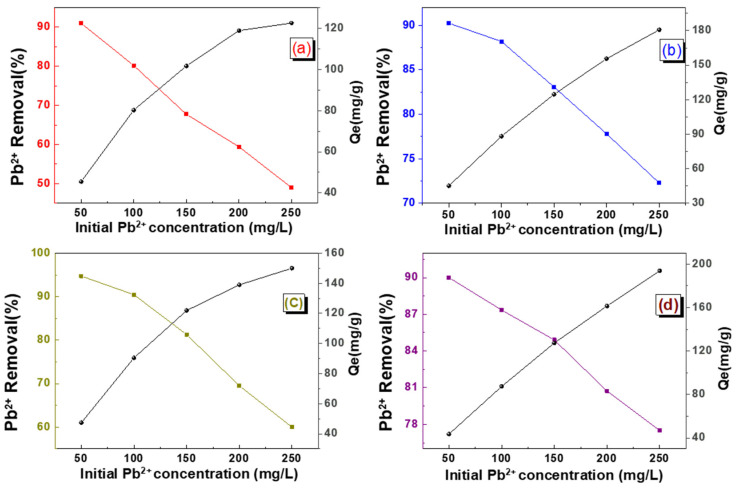
Effect of initial concentration on adsorption of Pb(II) ion (10–250 mg/L) with alginate/silica hybrid aerogels: SA-SiO_2_ (**a**), SA-SiO_2_-KH550 (**b**), SA-SiO_2_-KH580 (**c**), and SA-SiO_2_-KH590 (**d**).

By maintaining a constant solid-to-liquid ratio, we systematically investigated the removal efficiency and adsorption capacity across a range of dye concentrations (10–250 mg·L^−1^), as presented in Figure 6. The experimental results demonstrate an inverse correlation between lead ion removal efficiency and initial solution concentration. The alginate/silica hybrid aerogels exhibited superior adsorption performance for low-concentration lead ion solutions (<50 mg·L^−1^), suggesting their suitability for treating dilute heavy metal wastewater. The experimental maximum adsorption capacities of Pb^2+^ for SA-SiO_2_, SA-SiO_2_-KH550, SA-SiO_2_-KH580, and SA-SiO_2_-KH590 were 122.27 mg·g^−1^, 180.68 mg·g^−1^, 150.66 mg·g^−1^, and 193.73 mg·g^−1^, respectively. The observed concentration-dependent behavior can be attributed to the following: limited active sites becoming saturated at higher concentrations; enhanced mass transfer driving forces in dilute solutions; and potential competitive adsorption effects at elevated concentrations [29,30].

**Figure 7:**
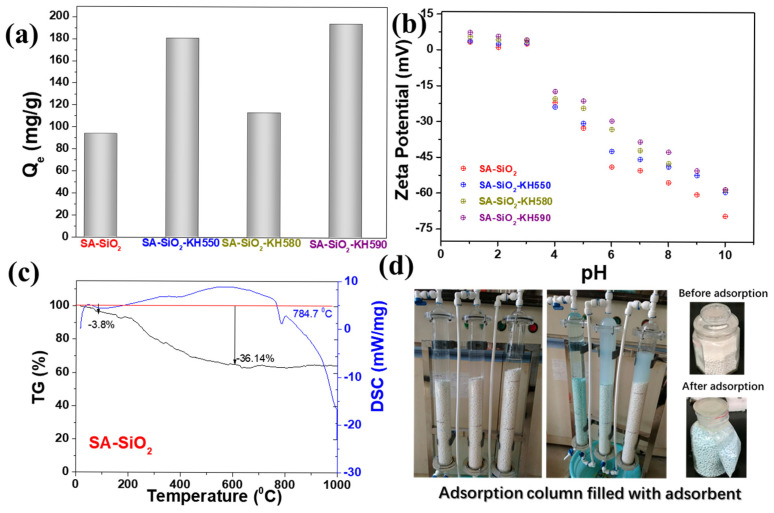
Adsorption capacity for Pb(II) ion (**a**) and zeta potentials (**b**) of alginate/silica hybrid aerogel beads; (**c**) thermogravimetric data of SA-SiO_2_; (**d**) adsorption column filled with adsorbent.

It can be seen from Figure 7 that the unfunctionalized SA-SiO_2_ aerogel beads demonstrated limited Pb^2^⁺ adsorption capacity (122.27 mg·g^−1^), despite their high specific surface area and hierarchical pore structure. This performance limitation stems from the absence of metal-coordinating functional groups in the pristine silica matrix, which restricts specific interactions with aqueous lead ions [31]. In contrast, functionalized SA-SiO_2_ aerogel beads exhibited significantly enhanced adsorption through surface functionalization effects [32], due to the introduction of amino or thiol groups, and had an increased number of active adsorption groups for chelating metal ions, thus improving the efficiency of the adsorbent for capturing metal ions [33]. For SA-SiO_2_-KH550, the incorporation of the aminopropyl group (-NH_2_) via silane coupling enabled Lewis acid base interactions [31], increasing its capacity to 180.68 mg·g^−1^. For SA-SiO_2_-KH580/KH590, thiol (-SH) functionalization provided soft Lewis basicity for strong Pb^2^⁺ coordination [34], achieving capacities of 150.66 and 193.73 mg·g^−1^, respectively. The enhanced performance correlated with the increased density of chelating sites [31], improved ion accessibility through optimized surface functionalization [32], and reduced interfacial energy between adsorbent and aqueous phase [35].

Surface charge analysis via zeta potential measurements revealed consistent surface potential across all alginate/silica hybrid aerogels (SA-SiO_2_), ranging from +10 mV to −60 mV over the pH spectrum, attributable to the ionization of Si–O bonds. As demonstrated in Figure 5, the maximum Pb(II) removal efficiencies occurred at pH 4–6 for all variants, correlating with their respective isoelectric points (PZC): SA-SiO_2_ at pH 3, SA-SiO_2_-KH550 at pH 4, and both SA-SiO_2_-KH580 and SA-SiO_2_-KH590 at pH 5 (Figure 7b). This pH-dependent behavior aligned with electrostatic interactions between negatively charged functional groups (–NH_2_ and –SH from silane modifiers) and hydrated Pb^2^⁺ cations. The alginate polymeric matrix primarily serves as a structural scaffold, while surface modifications (KH550/580/590) govern interfacial interactions. Enhanced Pb (II) uptake at pH 4–6 arose from two synergistic effects: (1) the optimal protonation states of amine/thiol groups favoring ligand metal coordination, and (2) the suppressed hydrolysis of Pb^2^⁺ below pH 7. Beyond pH 7, diminished adsorption correlated with the formation of hydrolyzed Pb species (e.g., Pb(OH)⁺, Pb₃(OH)₄^2^⁺, etc.), which exhibit reduced affinity for the aerogel surface. These findings emphasize the critical need to maintain operational pH below 7 for effective heavy metal sequestration [22]. Figure 7c shows the thermogravimetric data of the sample (SA-SiO_2_). It can be seen that the mass loss of the sample within 100 °C was only 3.8%, indicating that the water content in the sample was very low (3.8% weight loss below 100 °C). Meanwhile, the loss of the sample at 600 °C reached 36.14%, and the differential scanning calorimetry (DSC) peak was 784.7 °C. We filled the prepared hybrid aerogel beads in the adsorption column for adsorption experiments (Figure 7d). After adsorption, the aerogel beads remained in the state of microbeads all the time. This picture visually proved that the microbeads have certain mechanical properties after adsorption. Meanwhile, the video of manual compression of aerogel beads, as shown in Video S1, also verifies that this microsphere has certain mechanical properties.

The adsorption isotherm is a curve that reflects the relationship between the adsorption capacity and the equilibrium concentration after adsorption equilibrium [36]. The isotherm profile provides critical insights into adsorption mechanisms, including layer configuration and adsorbent adsorbate interaction modalities [21]. Two fundamental (Langmuir equation and the Freundlich equation) models were employed for system characterization [37]. The Langmuir theory assumes monolayer adsorption occurring at identical active sites with uniform adsorption energy. The Langmuir isothermal equation is expressed as follows:

(1) $$\frac{C_{e}}{Q_{e}}\;=\;\frac{1}{Q_{\max}K_{L}}\;+\;\frac{C_{e}}{Q_{\max}}$$

In the above formula, *Q_max_* represents the saturated adsorption capacity of adsorption (mg·g^−1^), *C_e_* represents the equilibrium concentration of the solution during the adsorption process (mg·L^−1^), *Q_e_* represents the adsorption capacity at equilibrium (mg·g^−1^), and K_L_ represents the affinity constant of the Langmuir isothermal equation (L·mg^−1^).

According to the Freundlich adsorption isothermal model theory, multi-layer adsorption occurs on heterogeneous surfaces [38]. The Freundlich adsorption isothermal equation is described as follows:

(2) $$Q_{e}\;=\;K_{F}C_{e}^{1/n}$$

In the above formula, *C_e_* represents the equilibrium concentration of the dye in the solution (mg L^−1^), and *Q_e_* represents the amount of dye adsorbed at adsorption equilibrium (mg/g). *K_F_* and 1/*n* are Freundlich’s characteristic constants, representing adsorption capacity and adsorption strength, respectively. If 1/*n* < 1, it indicates that the adsorption process occurs easily to proceed. If 1/*n* ≥ 2, it indicates that the adsorption process occurs with relatively difficulty.

**Figure 8:**
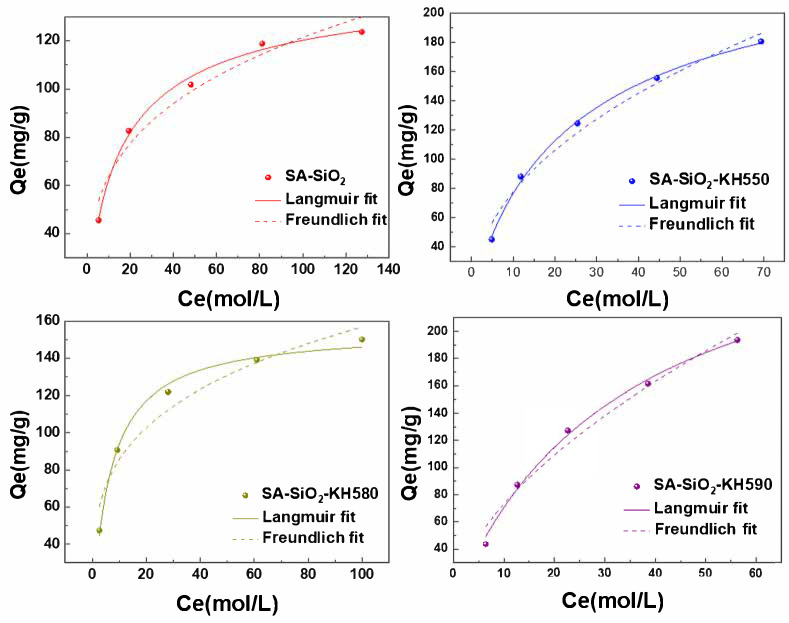
Adsorption isotherms of Pb(II) ion adsorbed on functional alginate/silica hybrid aerogel beads in solution.

The isotherms of lead ion adsorption by different thiol/amino-modified alginate/silica hybrid aerogel beads were fitted based on the data of the adsorption process (Figure 8), with the corresponding fitting parameters detailed in Table 2. It is evident from Table 2 that the Langmuir equation can describe the adsorption isotherms of lead ions on spherical aerogels quite well. Moreover, the theoretically calculated maximum adsorption capacities of lead ions by the hybrid spherical aerogel were 132.7 mg·g^−1^, 229.6 mg·g^−1^, 155.5 mg·g^−1^, and 208.3 mg·g^−1^, respectively, which deviated little from the data obtained from the experiment. Judging from the 1/n value obtained from the fitting of the Freundlich equation, the adsorption of heavy metal ions by the hybrid spherical gel occurred easily. From the perspective of adsorption state, the system adsorbed lead ions in a single-layer covering (Langmuir) mode. Specifically, SA-SiO_2_-KH550 showed the highest affinity, suggesting efficient amine Pb^2^⁺ coordination [21,37,38,39]. Furthermore, SA-SiO_2_-KH590 demonstrated the highest Q_max_, attributed to the optimal thiol group density and hierarchical porosity [26,37].

## 3. Conclusions

Alginate/silica hybrid aerogel beads were modified by using common silane coupling agents as modifiers. Several spherical aerogels such as SA-SiO_2_-KH550, SA-SiO_2_-KH580, and SA-SiO_2_-KH590 were successfully prepared by using the sharp pore–coagulation bath method and the ambient pressure drying method. The obtained SiO_2_ spherical aerogel was a milky white light solid. The size of the spherical aerogel was uniform. The material structure and microscopic morphology of the samples were studied using various characterization methods such as SXRD, SEM, and BET. The results show that this type of spherical aerogel had a nano-filamentous mesoporous structure. The heat treatment of hybrid aerogels yielded silicon carbon composite spherical aerogels and nano-filamentous silica aerogels, providing a certain significance to the synthesis of functional nanomaterials with different morphologies. The heavy metal adsorption experimental results show that the hybrid spherical aerogel had a good adsorption effect on heavy metals and maintained good sphericity, which has certain application prospects. The adsorption experiments show that the modified hybrid aerogel adsorbed lead ions well. A variety of spherical aerogels were prepared by using multiple molding processes, which solved the technical difficulties of easy cracking and difficult molding of aerogels during drying.

The modular architecture of alginate/silica hybrid aerogels, informed by recent advances in multivalent cationic cross linked alginate systems [40,41,42], presents significant potential for performance optimization and application diversification. This composite system demonstrates exceptional tunability through three primary design strategies: (1) the selection of appropriate crosslinking cations (e.g., Ca^2^⁺, Fe^3^⁺, Al^3^⁺), (2) the integration of functional additives (nanoparticles, polymers, or bioactive agents), and (3) synergistic combinations with complementary biopolymers. Such modular design principles establish alginate/silica hybrids as programmable material platforms with tailorable physicochemical properties. Emerging research suggests that the coordinated application of cation engineering, functional hybridization, and stimuli-responsive design could enable breakthroughs in three key domains: environmental remediation (heavy metal sequestration, catalytic filtration), biomedical applications (drug delivery scaffolds, tissue engineering matrices), and next-generation flexible electronics (compressible conductors, piezoresistive sensors).

## 4. Materials and Methods

### 4.1. Materials

Sodium alginate (CAS: 9005-38-3, M/G = 1:2), Calcium chloride (CaCl_2_), tetraethyl orthosilicate (TEOS), ethanol, 3-aminopropyl triethoxysilane (KH550), γ-methacryloxypropyl trimethoxy silane (KH580), and 3-mercaptopropyl trimethoxysilane (KH590) were purchased from Shanghai Macklin Biochemical Co., Ltd. (Shanghai, China). Deionized water was made from laboratory water purification equipment.

### 4.2. Synthesis of Alginate/Silica Binary Aerogel Beads (SA-SiO_2_)

Sodium alginate (1.5 g) was dissolved in deionized water (100 mL) under continuous magnetic stirring (500 rpm) for 1 h at ambient temperature. A 3 g quantity of CaCl_2_ was weighed and added to 100 mL of deionized water. This solution was stirred thoroughly for 12 h and kept aside. The above-mentioned sodium alginate solution was added drop by drop to the above-mentioned calcium chloride solution with a syringe pump with needle hole diameter of 0.7 mm to form sodium alginate hydrogel beads (the utilized programmable syringe pump had fixed parameters: (needle gauge) an inner diameter of 0.4 mm, an outer diameter of 0.7 mm, and a flow rate of 2 mL/min ± 0.1 mL). After aging for several hours, the prepared polymer spheres were washed with water three times, each time for 2 h, and the washing temperature was 40 °C. Then, the polymer spheres were soaked and aged in three times anhydrous ethanol at 40 °C, each time for 2 h. The polymer spheres were transferred to a mixed solution of tetraethyl orthosilicate (TEOS), ethanol, and water at 70 °C and soaked for 72 h. Finally, the wet beads were dried at room temperature for 24 h. The sample was named SA-SiO_2_. Moreover, the SA-SiO_2_ aerogel beads prepared above were placed in a tube furnace and calcined for 2 h under nitrogen (800 °C) and air atmosphere (600 °C), and they were named SA-SiO_2_-C and SA-SiO_2_-O, respectively.

### 4.3. Synthesis of Alginate/Silica Hybrid Aerogel Beads with Tunable Functional Surface

Hydrogel beads were obtained through the previous process. Then, the polymer beads were transferred to a mixed solution of silane coupling agents (KH-550, KH-580, and KH-590) and ethanol at 60 °C for immersion for 72 h (silane coupling agents were used directly and were not purified; a mixture of silane coupling agent and ethanol, in a volume ratio of 1:5, was used). Finally, the hybrid aerogel beads were washed in ethanol at 40 °C for 3 times, each time for 2 h. The hybrid beads were dried in an oven of 40 °C, and alginate/silica hybrid aerogel beads with tunable functional surface were obtained. The samples were named SA-SiO_2_-KH550, SA-SiO_2_-KH580, and SA-SiO_2_-KH590.

### 4.4. Standard Solution Curve

A 20 mL volume of Pb(II) solution of a certain concentration was prepared, and 20 mg of the adsorbent to be tested was added. This mixture was placed in a constant-temperature air bath shaker at 20 °C and shaken for 10 h (190 r/min), and samples were collected during the process. The samples were filtered and centrifugally separated, and the concentration of the remaining metal ions in the liquid phase was detected using a flame atomic absorption spectrophotometer. The adsorption rate η and adsorption capacity *Q_e_* of the aerogel sample for heavy metals in the aqueous solution were calculated. The formula for calculating of *Q_e_* is provided in Equation (3):

(3) $$Q_{e}=\frac{\left(C_{0}-C_{e}\right)V}{m}$$

Among them, *C_0_* (mg·L^−1^) and *Ce* (mg·L^−1^) are the initial and equilibrium concentrations of the heavy metal solution, respectively. *V* (L) and *m* (g) represent the volume of the solution and the amount of adsorbent used, respectively.

### 4.5. Material Characterizations

The small-angle X-ray diffraction (SXRD) patterns of beads were determined using a Bruker-AXS D8 Advance diffractometer equipped with Cu-Kα incident radiation (50 kV, 60 mA, a range of 0.5–10°). The phase purity of the beads was characterized using a Powder XRD diffraction instrument (Bruker AXS GmbH, karlsruhe, Germany) with CuKα radiation (λ = 1.5406 Å) at a scan rate of 7°/min in the 2θ range from 10° to 80°. Fourier transform infrared (FTIR) spectroscopy (Nicolet NEXUS 470, Thermo Scientific, Waltham, MA, USA) was performed using KBr discs. The Brunauer–Emmett–Teller (BET) specific surface area was obtained from the N_2_ adsorption–desorption analyses at 77 K (NOVA 2000, Quantachrome, Boynton Beach, FL, USA). The microstructure of the aerogel beads was assessed using field emission scanning electron microscope (SEM, S4800, Hitachi, Tokyo, Japan). The elemental analyzer (FlashSmart, ThermoFisher, Waltham, MA, USA) was used to analyze the mass percentages of the elements S, H, C, and N in the sample. The tapping densities and oil absorption capacity of the aerogel beads were also investigated as reported previously [8]. Zeta potential tests were performed on a Zetasizer Nano (ZEN5600, Malvern, Worcestershire, UK). The high-temperature resistance performance of the material samples was tested using a thermogravimetric analyzer produced by Netzsch (STA 449 F3 Jupiter, Wolverhampton, UK). The test temperature was room temperature ranging from 15 °C to 1000 °C. Nitrogen was used as the purging protective gas, an alumina crucible was used, and the sample mass was 5 mg.

## Figures and Tables

**Table 1:** Textural physical properties of alginate/silica hybrid aerogels.

Physical Property	SA-SiO_2_	SA-SiO_2_-C	SA-SiO_2_-O	SA-SiO_2_-KH550	SA-SiO_2_-KH580	SA-SiO_2_-KH590
Packing density (g/cm^3^)	0.160	0.155	0.148	0.177	0.171	0.174
Porosity (%)	91.67	92.67	92.44	90.67	90.64	89.94
Oil absorption (mL/g)	3.45	2.95	3.05	2.88	2.81	2.87
Water absorption (mL/g)	2.88	2.80	2.78	2.63	2.59	2.79
Shape and appearance	Spheroid Yellow	Spheroid Black	Spheroid Milky white	Spheroid Milky white	Spheroid Milky white	Spheroid Milky white
BET surface area (m^2^/g)	160.8	210.8	154.4	125.2	157.1	157.2
Pore volume (cm^3^/g)	0.54	0.75	0.95	0.36	0.51	0.64
Average pore diameter (nm)	6.6	4.3	3.4	3.1	3.4	3.4

**Table 2:** Constants of Langmuir and Freundlich isotherms for Pb(II) ion adsorption on functional alginate/silica hybrid aerogel beads.

Adsorption Isotherm Model	Constant	SA-SiO_2_	SA-SiO_2_-KH550	SA-SiO_2_-KH580	SA-SiO_2_-KH590
Langmuir equation	*R*	0.992	0.996	0.991	0.994
	*Q*_max_ (mg·g^−1^)	132.7	229.6	155.5	208.3
	*K*_L_(L·mg^−1^)	0.087	0.049	0.152	0.029
Freundlich equation	*R*	0.945	0.974	0.932	0.971
	*K*_F_ (mg·g^−1^)	33.38	27.45	46.71	19.32
	*1/n*	0.281	0.452	0.263	0.578

## Data Availability

The original contributions presented in the study are included in the article/Supplementary Material; further inquiries can be directed to the corresponding author.

## Supplementary Materials

The following supporting information can be downloaded at https://www.mdpi.com/article/10.3390/gels11060397/s1. Video S1: manual compression observation of hybrid aerogel bead.

## References

[1] Du A., Zhou B., Zhang Z., Shen J. (2013). A special material or a new state of matter: A review and reconsideration of the aerogel. Materials.

[2] Cai J., Liu S., Feng J., Kimura S., Wada M., Kuga S., Zhang L. (2012). Cellulose-silica nanocomposite aerogels by in-situ formation of silica in cellulose gel. Angew. Chem. Int. Ed..

[3] Jiménez-Saelices C., Seantier B., Cathala B., Grohens Y. (2017). Spray freeze-dried nanofibrillated cellulose aerogels with thermal superinsulating properties. Carbohydr. Polym..

[4] García-González C.A., Jin M., Gerth J., Alvarez-Lorenzo C., Smirnova I. (2015). Polysaccharide-based aerogel microspheres for oral drug delivery. Carbohydr. Polym..

[5] Hurtado A., Aljabali A.A.A., Mishra V., Tambuwala M.M., Serrano-Aroca Á. (2022). Alginate: Enhancement strategies for advanced applications. Int. J. Mol. Sci..

[6] Tordi P., Ridi F., Samorì P., Bonini M. (2025). Cation-Alginate complexes and their hydrogels: A powerful toolkit for the development of next-generation sustainable functional materials. Adv. Funct. Mater..

[7] Rahman M.M., Shahid M.A., Hossain M.T., Sheikh M.S., Rahman M.S., Uddin N., Rahim A., Khan R.A., Hossain I. (2024). Sources, extractions, and applications of alginate: A review. Discover Appl. Sci..

[8] Yu R., Shi Y., Yang D., Liu Y., Qu J., Yu Z.Z. (2017). Graphene oxide/chitosan aerogel microspheres with honeycomb-cobweb and radially oriented microchannel structures for broad-spectrum and rapid adsorption of water contaminants. ACS Appl. Mater. Interfaces.

[9] Zhang Y., Wang J., Zhang X. (2018). Surfactant-free synthesis of silica aerogel microspheres with hierarchically porous structure. J. Collid Interface Sci..

[10] Zong S., Wei W., Jiang Z., Yan Z., Zhu J., Xie J. (2015). Characterization and comparison of uniform hydrophilic/hydrophobic transparent silica aerogel beads: Skeleton strength and surface modification. RSC Adv..

[11] Wei W., Hu H., Yin S., Li Y., Ji X., Xie J. (2019). Rational fabrication of chitosan/alginate/silica ternary aerogel beads adsorbent with free separation. Micro Nano Lett..

[12] Han X.B., Li R., Miao P.P., Gao J., Hu G.W., Zhao Y., Chen T. (2022). Design, synthesis and adsorption evaluation of bio-based lignin/chitosan beads for congo red removal. Materials.

[13] Boccia A.C., Neagu M., Pulvirenti A. (2024). Bio-based aerogels for the removal of heavy metal ions and Oils from Water: Novel solutions for environmental remediation. Gels.

[14] Flores-Gómez J., Romero-Arellano V.H., Vazquez-Lepe M., Martínez-Gómez Á.d.J., Morales-Rivera J. (2023). Modeling and optimization of the adsorption of Cr (VI) in a chitosan-resole aerogel using response surface methodology. Gels.

[15] Xia S., Cui C., Li D., Wang L.J. (2025). Preparation of sodium alginate/quaternary ammonium-functionalized chitosan adsorbents: For the removal of high-molecular-weight invert sugar alkaline degradation products. Int. J Biol. Macromol..

[16] Elsayed I., Schueneman G.T., El-Giar E.M., Hassan E.B. (2023). Amino-functionalized cellulose nanofiber/lignosulfonate new aerogel adsorbent for the removal of dyes and heavy Metals from Wastewater. Gels.

[17] Iskandar M.A., Yahya E.B., Abdul Khalil H.P.S., Rahman A.A., Ismail M.A. (2022). Recent Progress in modification strategies of nanocellulose-based aerogels for oil absorption application. Polymers.

[18] Sadat Fazel S., Jonoobi M., Pourtahmasi K., Sepahvand S., Ashori A. (2024). Enhancing the oil adsorption properties of cellulose nanofiber aerogels through chemical modification. J. Polym. Environ..

[19] Fan K., Zhang T., Xiao S., He H., Yang J., Qin Z. (2022). Preparation and adsorption performance of functionalization cellulose-based composite aerogel. Int. J Biol. Macromol..

[20] Hou Y., Zhong X., Ding Y., Zhang S., Shi F., Hu J. (2020). Alginate-based aerogels with double catalytic activity sites and high mechanical strength. Carbohydr. Polym..

[21] Boateng I.D., Yang X.M., Yin H., Liu W. (2024). Separation and purification of polyprenols from Ginkgo biloba leaves by silver ion anchored on imidazole-based ionic liquid functionalized mesoporous MCM-41 sorbent. Food Chem..

[22] Singh S., Basu H., Bassan M.K.T., Singhal R.K. (2022). Thiol functionalised silica microsphere loaded polymeric hydrogel: Development of a novel hybrid sorbent for removal of lead and cadmium. Chemosphere.

[23] Wang B., Li D., Tang M., Ma H., Gui Y., Tian X., Quan F., Song X., Xia Y. (2018). Alginate-based hierarchical porous carbon aerogel for high-performance supercapacitors. J. Alloys Compd..

[24] Ji D., Park J.M., Oh M.S., Nguyen T.L., Shin H., Kim J.S., Kim D., Park H.S., Kim J. (2022). Superstrong, superstiff, and conductive alginate hydrogels. Nat. Commun..

[25] Fajardo A.R., Silva M.B., Lopes L.C., Piai J.F., Rubira A.F., Muniz E.C. (2012). Hydrogel based on an alginate–Ca^2+^/chondroitin sulfate matrix as a potential colon-specific drug delivery system. RSC Adv..

[26] Tanimu A., Ganiyu S.A., Kozhevnikov I., Alhooshani K. (2021). In-situ activation of NiMo catalyst based on support surface-bound thiols: A green approach to catalyst sulfidation and improved activity. Arab. J. Chem..

[27] Zhang Y., Feng T., Ni X., Xia J., Suo H., Yan L., Zou B. (2024). Immobilized lipase based on SBA-15 adsorption and gel embedding for catalytic synthesis of isoamyl acetate. Food Biosci..

[28] Zhang D., Wang C., Bao Q., Zheng J., Deng D., Duan Y., Shen L. (2018). The physicochemical characterization, equilibrium, and kinetics of heavy metal ions adsorption from aqueous solution by arrowhead plant (*Sagittaria trifolia* L.) stalk. J. Food Biochem..

[29] Liu H., Li P., Zhang T., Zhu Y., Qiu F. (2020). Fabrication of recyclable magnetic double-base aerogel with waste bioresource bagasse as the source of fiber for the enhanced removal of chromium ions from aqueous solution. Food Bioprod. Process..

[30] Jiang C., Wang X., Hou B., Hao C., Li X., Wu J. (2020). Construction of a lignosulfonate–lysine hydrogel for the adsorption of heavy metal ions. J. Agric. Food Chem..

[31] Fan X., Peng L., Wang X., Han S., Yang L., Wang H., Hao C. (2022). Efficient capture of lead ion and methylene blue by functionalized biomass carbon-based adsorbent for wastewater treatment. Ind. Crop. Prod..

[32] Han S., Xie H., Zhang L., Wang X., Zhong Y., Shen Y., Wang H., Hao C. (2023). High-performance polyethylenimine-functionalized lignin/silica porous composite microsphere for the removal of hexavalent chromium, phosphate and Congo red from aqueous solutions. Ind. Crop. Prod..

[33] Shao Z., Xing C., Xue M., Fang Y., Li P. (2023). Selective removal of Pb (II) from yellow rice wine using magnetic carbon-based adsorbent. J. Sci. Food Agric..

[34] Wang X., Hu X., Zhai X., Huang X., Li Z., Zou X., Shi J. (2024). A simple and sensitive electrochemical sensing based on amine-functionalized metal–organic framework and polypyrrole composite for detection of lead ions in meat samples. J. Food Meas. Charact..

[35] Wang X., Xu Y., Li Y., Li Y., Li Z., Zhang W., Zou X., Shi J., Huang X., Liu C. (2021). Rapid detection of cadmium ions in meat by a multi-walled carbon nanotubes enhanced metal-organic framework modified electrochemical sensor. Food Chem..

[36] Chen Y., Zhang W., Zhao T., Li F., Zhang M., Li J., Zou Y., Wang W., Cobbina S.J., Wu X. (2016). Adsorption properties of macroporous adsorbent resins for separation of anthocyanins from mulberry. Food Chem..

[37] Liu Y., Jing Z., Zhang T., Chen Q., Qiu F., Peng Y., Tang S. (2018). Fabrication of functional biomass carbon aerogels derived from sisal fibers for application in selenium extraction. Food Bioprod. Process..

[38] Zhang J., Fu W. (2020). Sponge effect of aerated concrete on phosphorus adsorption-desorption from agricultural drainage water in rainfall. Soil Water Res..

[39] Buema G., Segneanu A.-E., Herea D.-D., Grozescu I. (2024). Gels for water remediation: Current research and perspectives. Gels.

[40] Tordi P., Gelli R., Tamantini S., Bonini M. (2025). Alginate crosslinking beyond calcium: Unlocking the potential of a range of divalent cations for fiber formation. Int. J. Biol. Macromol..

[41] Choi I., Lee Y., Lyu J.S., Lee J.S., Han J. (2022). Characterization of ionically crosslinked alginate films: Effect of different anion-based metal cations on the improvement of water-resistant properties. Food Hydrocoll..

[42] Malektaj H., Drozdov A.D., deClaville Christiansen J. (2023). Mechanical properties of alginate hydrogels cross-linked with multivalent cations. Polymers.

